# Considerations on the Relativity of Quantum Irrealism

**DOI:** 10.3390/e25040603

**Published:** 2023-04-01

**Authors:** Nicholas G. Engelbert, Renato M. Angelo

**Affiliations:** Department of Physics, Federal University of Paraná, P.O. Box 19044, Curitiba 81531-980, Paraná, Brazil

**Keywords:** quantum irrealism, Wigner rotation, special relativity

## Abstract

The study of quantum resources in the relativistic limit has attracted attention over the last couple of decades, mostly due to the observation that the spin-momentum entanglement is not Lorentz covariant. In this work, we take the investigations of relativistic quantum information a step further, bringing the foundational question of realism to the discussion. In particular, we examine whether Lorentz boosts can affect quantum irrealism—an instance related to the violations imposed by quantum mechanics onto a certain notion of realism. To this end, we adopt as a theoretical platform a model of a relativistic particle traveling through a Mach–Zehnder interferometer. We then compare the quantum irrealism assessed from two different inertial frames in relative motion. In consonance with recent findings in the context of quantum reference frames, our results suggest that the notion of physical realism is not absolute.

## 1. Introduction

Denying action at a distance and superdeterminism falsifies *realism*—the hypothesis that physical quantities take on definite values prior to observation. This is an important conclusion emerging from a long-lasting debate initiated by Einstein, Podolsky, and Rosen (EPR) through their famous 1935 paper [1]. By rejecting the possibility that two space-like separated particles may influence each other (the locality hypothesis), EPR conceived a scenario whereby incompatible observables should be simultaneous elements of the physical reality. Unable to predict definite values for such observables, quantum mechanics was then claimed to be an incomplete theory. This position was soon confronted by Bohr [2], who resorted to the complementarity principle to argue that incompatible observables could only become elements of reality through mutually exclusive experimental arrangements, never simultaneously. Adopting a different perspective, Bohm proposes in 1952 to restore realism by the introduction of deterministic trajectories [3,4], with the caveat of accepting nonlocality. Years later, Bell showed that any eventual hidden-variable theory intended to replicate the predictions of quantum mechanics would be necessarily nonlocal [5]. Numerous loophole-free experiments [6,7,8,9,10,11] have now confirmed that nature is at odd with Bell’s hypothesis of local causality (sometimes called local realism hypothesis [8,11]).

Long gone the time of Bell and EPR, foundational questions regarding realism still provoke interest in the quantum research community. In search for a notion of realism fundamentally disconnected from any locality hypothesis, Bilobran and Angelo introduced both a criterion of realism and a quantifier of quantum irrealism (the antithesis of realism) [12]. Their framework has recently been experimentally tested in a nuclear magnetic resonance platform and Bohr’s idea that one cannot find simultaneous elements of reality within the same setting has been confirmed [13]. In a slightly different vein but still related to realism, the Wigner friend thought experiment [14], arguably the most stripped-down statement of the measurement problem, has been revisited. Frauchiger and Renner showed that quantum mechanics cannot consistently describe the use of itself [15]. Brukner devised a no-go theorem regarding observer-independent facts [16]. A different no-go theorem due to Bong et al. showed that nature cannot simultaneously satisfy the hypothesis of no-superdeterminism, locality, and “absoluteness of observed events” [17].

An essential ingredient for the majority of the aforementioned works is quantum entanglement [18], a key element distinguishing quantum mechanics from the classical worldview. It has been framed as a quantum resource [19], responsible for the success of tasks such as cryptography [20] and teleportation [21,22]. Given the ubiquitous relevance of this resource in most quantum mechanical systems, the study of entanglement has gained traction among areas as diverse as cosmology [23], quantum biology [24], and notably, special relativity [25]. In this latter realm, one of the most surprising and impactful results was that a Lorentz boost (i.e., a Lorentz transformation onto a moving frame) may alter the entanglement between the spin and momentum of a single relativistic particle. This was first discussed in a seminal paper by Peres et al. [26], which sparked a multitude of related developments exploring and further confirming that result, not only in the single-particle case [27,28,29,30,31,32,33,34,35] but also for two or more particles [36,37,38,39,40,41,42,43,44,45]. As a natural sequel to these works, some discussions emerged concerning the boost effects on Bell inequality violations [46,47,48,49,50]. Within this context, many alternative propositions came for an adequate relativistic spin observable [47,51,52,53] and relativistic reduced spin density matrix [32,54]. At the heart of the strange entangling effects of the boost is the Wigner rotation [55,56,57]. It is a well-known (albeit awkward) consequence of special relativity that the composition of two non-collinear Lorentz boosts is equivalent to a boost plus a rotation (the Wigner rotation). This effect plays a fundamental role in the transformation law of single-particle states.

Now, since entanglement and quantum coherence are (frame-dependent) quantum resources forbidding one to support (local) realism, it is natural to ask whether existing realistic models respect Lorentz covariance. Despite the aforementioned advancements in relativistic quantum mechanics, the understanding of this specific aspect has remained elusive. Recently, within the framework of quantum reference frames [58], it was shown that the elements of reality are not covariant under Galilei transformations [59]. The present work aims to extend this line of inquiry to the domain of special relativity, by addressing the question of whether elements of reality are absolute notions under Lorentz boosts. To this end, we will employ as a theoretical platform of investigation the Mach–Zehnder interferometer (MZI). By use of the realism framework proposed by Bilobran and Angelo, we analyze how the *irreality* (quantum indefiniteness) of spin is affected by a Lorentz boost as a particle travels an MZI. In addition, we investigate the role of spin-momentum entanglement in the breakdown of realism. We find, in agreement with the results reported for the context of Galilei relativity [59], that physical realism is not frame-independent. Crucial for deepening the understanding of foundational aspects in the interface between quantum mechanics and special relativity, our results show that Lorentz-boosted observers do not generally agree on the adequacy of realistic interpretations to base their experiences.

## 2. Methods

In what follows, we briefly review the theoretical tools employed in this work.

### 2.1. Quantum Irrealism

In 2015, a criterion of physical realism and a quantifier of its violation was put forward [12] which have seen fruitful applications in several (theoretical and experimental) foundational investigations [60,61,62,63,64,65,66]. The basic premise of the framework is that in a realistic theory, the role of measurement is merely revealing an already installed *element of reality*—a well-defined value of which the observer was previously ignorant. It then follows that if the measurement outcome is kept secret, then the probing has been entirely innocuous and no change in the state of knowledge can take place whatsoever. To formalize this idea, consider a preparation ρ on the space $H_{A}⊗H_{B}$, where $H_{B}$ is possibly multipartite. If a discrete spectrum observable $A=\sum_{i}a_{i}A_{i}$ acting on $H_{A}$ with projectors $A_{i}$ is measured but the measurement outcome is not revealed, then the post-measurement state is given by the following CPTP unital map:

(1) $$\begin{array}{c} \Phi_{A}\left(\rho \right)=\sum_{i}\left(A_{i}⊗{𝟙}_{B}\right)\;\rho \;\left(A_{i}⊗{𝟙}_{B}\right). \end{array}$$

With that, the authors of Ref. [12] propose that the observable *A* will be an element of reality when their criterion of realism,

(2) $$\begin{array}{c} \Phi_{A}\left(\rho \right)=\rho , \end{array}$$

is satisfied by a preparation ρ. The aforementioned premise materializes through the above equation in the sense that the measurement probability $p\left(b_{j}\right)=\mathrm{Tr}\left(B_{j}\rho \right)$ for any observable $B=\sum_{i}b_{j}B_{j}$, given a preparation satisfying Equation (2), is never altered when the element of reality *A* is non-selectively measured, that is, $\mathrm{Tr}\left[B_{j}\Phi_{A}\left(\rho \right)\right]=\mathrm{Tr}\left(B_{j}\rho \right)$. This is taken as the defining feature of a realistic theory (of which, classical-statistical mechanics is an example).

The authors of Ref. [12] then introduced the so-called *irreality*

(3) $$\begin{array}{c} I_{A}\left(\rho \right):=S\left(\rho ||\Phi_{A}\left(\rho \right)\right)=S\left(\Phi_{A}\left(\rho \right)\right)-S\left(\rho \right), \end{array}$$

a measure of “how far” *A* is from being an element of reality. In other words, $I_{A}\left(\rho \right)$ measures the degree to which the hypothesis of *A*-realism, as given by Equation (2), is violated by a preparation ρ. Here, $S\left(\rho \right)=-\mathrm{Tr}\left(\rho \log_{2}\rho \right)$ is the von Neumann entropy, measured in bits, and $S\left(\rho ||\sigma \right)=\mathrm{Tr}\left[\rho \left(\log_{2}\rho -\log_{2}\sigma \right)\right]$ is the quantum relative entropy. It readily follows from the properties of the relative entropy that $I_{A}\left(\rho \right)$ is nonnegative and vanishes if the preparation is an *A*-reality state (i.e., a state satisfying Equation (2)). In addition, irreality has recently been formally framed as a quantum resource [67]. Furthermore, noteworthy is the fact that the quantifier (3) has been assigned an axiomatic perspective and has received different interpretations in the literature (see [66] and references therein).

Seen as a bipartite generalization of the well-known dephasing map from the resource theory of coherence [68], $\Phi_{A}\left(\rho \right)$ has the effect of removing not only *A*-coherence from ρ, but also quantum correlations between the two degrees of freedom represented by the spaces $H_{A}$ and $H_{B}$. In effect, it can be shown that the irreality admits the following decomposition:

(4) $$\begin{array}{c} I_{A}\left(\rho \right)=C_{A}\left(\rho_{A}\right)+D_{A}\left(\rho \right), \end{array}$$

where $D_{A}\left(\rho \right)=I_{A:B}\left(\rho \right)-I_{A:B}\left(\Phi_{A}\left(\rho \right)\right)$ is the one way quantum discord of the measurement *A* and $C_{A}\left(\rho_{A}\right)=S\left(\Phi_{A}\left(\rho_{A}\right)\right)-S\left(\rho_{A}\right)$ is the relative entropy of *A*-coherence [68]. The decomposition above is the fundamental inspiration for the present investigation, for it tells us that quantum correlations violate realism. Since entanglement is known to vary upon Lorentz boots, it is natural to ask how realism is violated in different reference frames.

### 2.2. Lorentz Boosts and Wigner Rotations

We now recap the essential elements of the relativistic quantum mechanics of single-particle systems, in a review mostly based upon [69]. The usual description of single-particle states in relativistic quantum mechanics relies on eigenstates |p,σ〉 of the four-momentum operator and some spin component, which we take to be $S_{z}$. An arbitrary four-momentum *p* can be related to the rest four-momentum k=(mc,0,0,0) through a Lorentz boost L(p), such that p=L(p)k, or in terms of quantum states, $|p,\sigma \rangle =U\left(L(p)\right)|k,\sigma \rangle$, where $U\left(L(p)\right)$ is the unitary representation of L(p). The transformation of these states under an arbitrary Lorentz boost Λ is given by

(5) $$\begin{array}{c} U\left(\Lambda \right)|p,\sigma \rangle =U\left(\Lambda \right)\;U\left(L(p)\right)|k,\sigma \rangle . \end{array}$$

It is a well-known consequence of special relativity that the composition of two non-collinear boosts is equal to a single boost plus a rotation *R*—the so-called Wigner rotation. Thus, if the direction of the boost Λ is not the same as the particle’s three-momentum p, then it follows that ΛL(p)=L(Λp)R(Ω), whose unitary representation reads

(6) $$\begin{array}{cc} U(\Lambda )|p,\sigma \rangle & =U(L(\Lambda p))\;U\left(R(\Omega )\right)|k,\sigma \rangle \\ & =U(R(\Omega ))|\Lambda p,\sigma \rangle \\ & =\sum_{\sigma^{\prime }}D_{\sigma \sigma^{\prime }}|\Lambda p,\sigma^{\prime }\rangle , \end{array}$$

where D≡U(R(Ω)) is the unitary representation of the Wigner rotation. For a spin $-\frac{1}{2}$ particle, this reduces to the usual rotation matrix of a qubit:

(7) $$\begin{array}{c} D=e^{-i\;\hat{n}\cdot \sigma \;\Omega /2}=\cos\left(\frac{\Omega }{2}\right)-i\sin\left(\frac{\Omega }{2}\right)\;\hat{n}\cdot \sigma . \end{array}$$

The rotation axis $\hat{n}$ and the angle Ω are well documented in the literature surrounding the Wigner rotation; the former is always mutually orthogonal to the boosts being composed, which in this case means

(8) $$\begin{array}{c} \hat{n}=\frac{v\times p}{\left||v\times p|\right|}, \end{array}$$

with v being the velocity associated with the boost Λ. As for the angle, in the particular case of a boost perpendicular to the particle’s movement, the following formula holds:

(9) $$\begin{array}{c} \cos\Omega =\frac{\left(\gamma_{\Lambda }+1\right)\left(\gamma_{p}+1\right)}{\gamma_{\Lambda }\gamma_{p}+1}-1, \end{array}$$

where $\gamma_{\Lambda }$ and $\gamma_{p}$ are the Lorentz factors for the boosts Λ and L(p), respectively. For the boost L(p) from the particle’s rest frame, one has $\gamma_{p}=E_{p}/mc^{2}$, with $E_{p}=\sqrt{p^{2}c^{2}+m^{2}c^{4}}$, such that equation above can be further simplified to

(10) $$\begin{array}{c} \cos\Omega =\frac{E_{p}+\gamma_{\Lambda }mc^{2}}{\gamma_{\Lambda }E_{p}+mc^{2}}. \end{array}$$

This means that Ω is a monotonically increasing function of the boost speed β≡||v||c and of the momentum magnitude ||p||. With Equation (6) and the subsequent formulas, any single-particle state can be transformed under U(Λ) to a state as seen by the boosted frame. (The state transformation employed here implies that we are using the active picture, as opposed to the passive picture in which the observables are transformed via $A\to U^{†}\left(\Lambda \right)\;A\;U\left(\Lambda \right)$ [59,70]).

For a simple example of how the transformation (6) may affect entanglement between spin and momentum, consider the state $|\psi \rangle =\left(|p_{1},\sigma \rangle +|p_{2},\sigma \rangle \right)/\sqrt{2}$ of a single relativistic fermion. This state is obviously separable. Equation (6) tells us that under a generic Lorentz boost the state transforms to $|\psi^{\prime }\rangle =\left(|\Lambda p_{1},\sigma_{1}\rangle +|\Lambda p_{2},\sigma_{2}\rangle \right)/\sqrt{2}$, where $|\sigma_{1}\rangle \equiv D_{1}|\sigma \rangle$ and $|\sigma_{2}\rangle \equiv D_{2}|\sigma \rangle$ are spin states that underwent different Wigner rotations ($D_{j}$ is associated with momentum $p_{j}$, with j∈{1,2}). Thus, a state which is initially separable in a given reference frame may be seen as having spin-momentum entanglement in a different inertial frame. Although it is still debatable whether and how such entanglement can be experimentally validated [30], the theoretical position commonly adopted in the literature is that this type of entanglement is meaningful.

## 3. Results

We now consider a relativistic particle passing a Mach–Zehnder interferometer (MZI) while observed from two distinct reference frames in relative motion. Specifically, we want to investigate whether physical reality is absolute, that is, if it manifests itself in the same way in all Lorentz frames. To this end, we compute the irreality, as defined in the Methods section, for both spin and momentum, in the two frames of reference.

**Figure 1:**
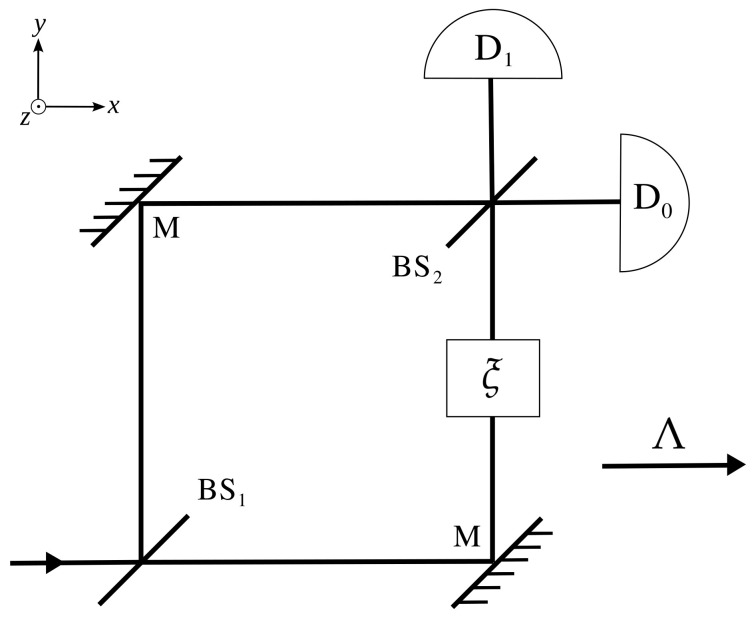
The Mach–Zehnder interferometer (MZI): an incoming electron reaches a beam-splitter (BS_1_) which puts it into a superposition of paths. Upon reflection in the mirrors (M) and recombination in the second beam-splitter (BS_2_), the probability of detection in either detectors D_0_ or D_1_ is dictated by the relative phase ξ. Furthermore, depicted is the direction of the boost Λ.

The whole setup is depicted in Figure 1. The first beam-splitter (BS_1_) creates a superposition of paths for the incoming particle beam. Then, two mirrors (M) redirect the paths aiming at closing a circuit, while the second beam-splitter (BS_2_) recombines the two superposed beams, making them interfere. Two detectors (D_0,1_) are placed at the two output ports of the second beam-splitter, and the probability of detection in each of them depends on the relative phase ξ (a controllable parameter) between the two superposed beams.

To make the boost’s entangling effects explicit, we shall cast the description of the MZI in terms of four-momentum eigenstates. We assume that the MZI is aligned with the *x* and *y* axes of the lab frame, so that while propagating in the horizontal arms the particle’s four-momentum is $p_{H}=\left(E_{p}/c,p,0,0\right)$, while in the vertical arms, it is given by $p_{V}=\left(E_{p}/c,0,p,0\right)$. The trajectory over the MZI of an incoming horizontal beam, up until before the second beam-splitter, is formally described by the sequence of maps

(11) $$\begin{array}{c} |p_{H}\rangle \overset{\mathrm{BS}{}_{1}}{\to }\frac{|p_{H}\rangle +i|p_{V}\rangle }{\sqrt{2}}\overset{M}{\to }\frac{|p_{H}\rangle -i|p_{V}\rangle }{\sqrt{2}}\overset{\xi }{\to }\frac{|p_{H}\rangle -ie^{i\xi }|p_{V}\rangle }{\sqrt{2}}, \end{array}$$

with the mirror action being given by $|p_{V(H)}\rangle \overset{M}{\to }i|p_{H(V)}\rangle$. The second beam-splitter then creates interference between the two paths, resulting in the final state

(12) $$\begin{array}{c} |\psi_{f}\rangle =\left[\cos\left(\frac{\xi }{2}\right)|p_{H}\rangle +\sin\left(\frac{\xi }{2}\right)|p_{V}\rangle \right]|\Phi \rangle , \end{array}$$

where the phase ξ controls the probability of observing the particle at each detector. We denote the particle’s spin state by |Φ〉, and take it to be an eigenstate of $\sigma_{\hat{n}}=\sigma \cdot \hat{n}$ for an arbitrary direction defined by the unit vector $\hat{n}=\left(\sin\theta \;\cos\phi ,\sin\theta \;\sin\phi ,\cos\theta \right)$. The MZI is seen to be completely insensitive to spin, i.e., the state remains |Φ〉 for the entire trajectory, which also means that $\sigma_{\hat{n}}$ is always an element of reality in the lab frame.

### 3.1. MZI from a Boosted Frame

We now move on to consider the same experiment viewed from a reference frame $S^{\prime }$ moving with velocity $v=\beta c\hat{x}$ relative to the lab frame. The horizontal and vertical momenta seen from this frame are $\pi_{H}\equiv \Lambda p_{H}$ and $\pi_{V}\equiv \Lambda p_{V}$, which, for a boost in the *x* direction, are given by

(13) $$\begin{array}{c} \begin{array}{cc} \pi_{H} & =\left(\pi_{H}^{0},\pi_{H}^{x},\pi_{H}^{y},\pi_{H}^{z}\right)=\frac{\gamma }{c}\left(E_{p}-\beta cp,pc-\beta E_{p},0,0\right),\\ \pi_{V} & =\left(\pi_{V}^{0},\pi_{V}^{x},\pi_{V}^{y},\pi_{V}^{z}\right)=\frac{\gamma }{c}\left(E_{p},-\beta E_{p},cp/\gamma ,0\right), \end{array} \end{array}$$

where $\gamma ={\left(1-\beta^{2}\right)}^{-1/2}$. The correspondence between the states on each frame is then simply obtained through Equation (6):

(14) $$\begin{array}{c} \begin{array}{cc} |p_{H}\rangle |\Phi \rangle & \overset{\Lambda }{\to }|\pi_{H}\rangle |\Phi \rangle ,\\ |p_{V}\rangle |\Phi \rangle & \overset{\Lambda }{\to }|\pi_{V}\rangle |\Phi_{R}\rangle , \end{array} \end{array}$$

where $|\Phi_{R}\rangle \equiv R_{\hat{z}}\left(\Omega \right)|\Phi \rangle$ is the Wigner-rotated spin state, $R_{\hat{z}}\left(\Omega \right)$ denotes a rotation around the axis $\hat{v}\times \hat{p}=\hat{z}$, and

(15) $$\begin{array}{c} \begin{array}{cc} & |\Phi \rangle =\cos\left(\frac{\theta }{2}\right)|0\rangle +e^{i\phi }\sin\left(\frac{\theta }{2}\right)|1\rangle ,\\ & |\Phi_{R}\rangle =\cos\left(\frac{\theta }{2}\right)|0\rangle +e^{i(\phi +\Omega )}\sin\left(\frac{\theta }{2}\right)|1\rangle . \end{array} \end{array}$$

Notice that no Wigner rotation occurs in the horizontal arms of the MZI because the boost is parallel to the momentum in that case, so the spin state remains unaltered. The rotation around the *z* axis adds a relative phase Ω (the Wigner rotation angle) to the spin state. Applying the transformations (14) to the state (12) yields the transformed output state

(16) $$\begin{array}{c} \;|\psi_{f}^{\prime }\rangle =U\left(\Lambda \right)|\psi_{f}\rangle =\cos\left(\frac{\xi }{2}\right)|\pi_{H}\rangle |\Phi \rangle +\sin\left(\frac{\xi }{2}\right)|\pi_{V}\rangle |\Phi_{R}\rangle . \end{array}$$

**Figure 2:**
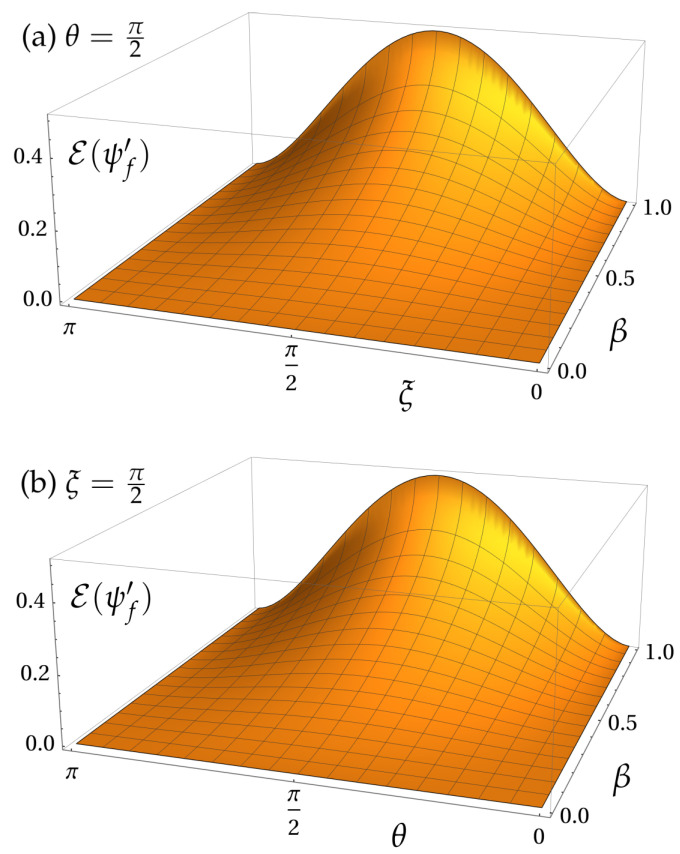
Entropy of entanglement $E(\psi_{f}^{\prime })$ between spin and momentum for the output state of the MZI in the boosted reference frame $S^{\prime }$. (**a**) $E(\psi_{f}^{\prime })$ is plotted as a function of the phase ξ and boost velocity β, while the spin state is fixed in the equator of the Bloch sphere (θ=π/2). (**b**) The phase ξ is held constant (ξ=π/2) and $E(\psi_{f}^{\prime })$ is plotted as function of θ and β. Entanglement is shown to typically increase with the boost velocity, except for very specific geometric configurations. Throughout this paper, all numerical simulations were made with $\gamma_{p}=5$.

The immediate consequence of the transformation law (14), which can be promptly noticed in the state above, is that whenever there is a superposition of different momenta, which occurs when ξ≠nπ for n∈N, there will be entanglement between spin and momentum. The amount $E(\psi_{f}^{\prime })$ of spin-momentum entanglement encoded in the pure state $\psi_{f}^{\prime }\equiv |\psi_{f}^{\prime }\rangle \;\langle \psi_{f}^{\prime }|$ can be computed through the von Neumann entropy of the spin reduced state $\rho_{S}^{\prime }=\cos^{2}\left(\xi /2\right)|\Phi \rangle \;\langle \Phi |+\sin^{2}\left(\xi /2\right)|\Phi_{R}\rangle \;\langle \Phi_{R}|$. The result reads

(17) $$\begin{array}{c} E\left(\psi_{f}^{\prime }\right)=S\left(\rho_{S}^{\prime }\right)=h\left(\frac{1}{2}+\frac{1}{8}\sqrt{2\lambda }\;\right), \end{array}$$

where $h\left(u\right)\equiv -u\log_{2}u-\left(1-u\right)\log_{2}\left(1-u\right)$ is the binary entropy, for any real parameter u∈[0,1], and $\lambda \equiv 7+\cos\Omega +2\left[\cos\left(2\xi \right)+2\cos\left(2\theta \right)\sin^{2}\xi \right]\sin^{2}\left(\frac{\Omega }{2}\right)$. Figure 2 shows $E(\psi_{f}^{\prime })$ as a function of the boost velocity β (which controls the angle Ω), the spin polar angle θ, and the phase ξ. The values ξ=0 and ξ=π, respectively, imply 100% of detection rate on detectors D_0_ and D_1_, and no momentum superposition in the output state. Hence, no entanglement is generated in these cases. Another less obvious instance in which there is no entanglement is when θ∈{0,π} (i.e., when the spin state is either |0〉 or |1〉). That is because the Wigner rotation is around the *z*-axis, so it leaves $\sigma_{z}$ eigenstates invariant. The values $\xi =\frac{\pi }{2}$ and $\theta =\frac{\pi }{2}$ maximize the entanglement effect. For these values, the entropy of entanglement reduces to $E\left(\psi_{f}^{\prime }\right)=h\left[\;\cos^{2}\left(\Omega /4\right)\right]$, which increases monotonically with Ω. These results just check the widely-spread mantra that, provided one can measure spin separately from momentum, then the spin-momentum entanglement will be found to be a boost-dependent resource.

### 3.2. Boost Effects on Spin Irreality

We now address the fundamental question of whether physical reality, as defined by Bilobran and Angelo’s work [12], is absolute or relative. For this, we will employ the criterion of realism (2) and the irreality quantifier (3) to investigate the ontological status of spin and momentum for a particle traveling through the MZI. The first thing to notice is that the spin component $\sigma_{\hat{n}}=\hat{n}\cdot \sigma$ is always an element of reality in the lab frame. That is, when β=0, we have $\Phi_{\sigma_{\hat{n}}}\left(\rho \right)=\rho$ and then

(18) $$\begin{array}{c} I_{\sigma_{\hat{n}}}\left(\psi_{f}\right)=0, \end{array}$$

for $\psi_{f}\equiv |\psi_{f}\rangle \;\langle \psi_{f}|$. (This is true for the output state as well as for all the intermediate states along the MZI). For the output state in the moving frame, $\psi_{f}^{\prime }$, the irreality of $\sigma_{\hat{n}}$ increases with the Wigner rotation angle. In terms of binary entropy, we have

(19) $$\begin{array}{c} I_{\sigma_{\hat{n}}}\left(\psi_{f}^{\prime }\right)=h\left[\sin^{2}\;\theta \;\;\sin^{2}\;\left(\frac{\xi }{2}\right)\;\sin^{2}\;\left(\frac{\Omega }{2}\right)\right]. \end{array}$$

**Figure 3:**
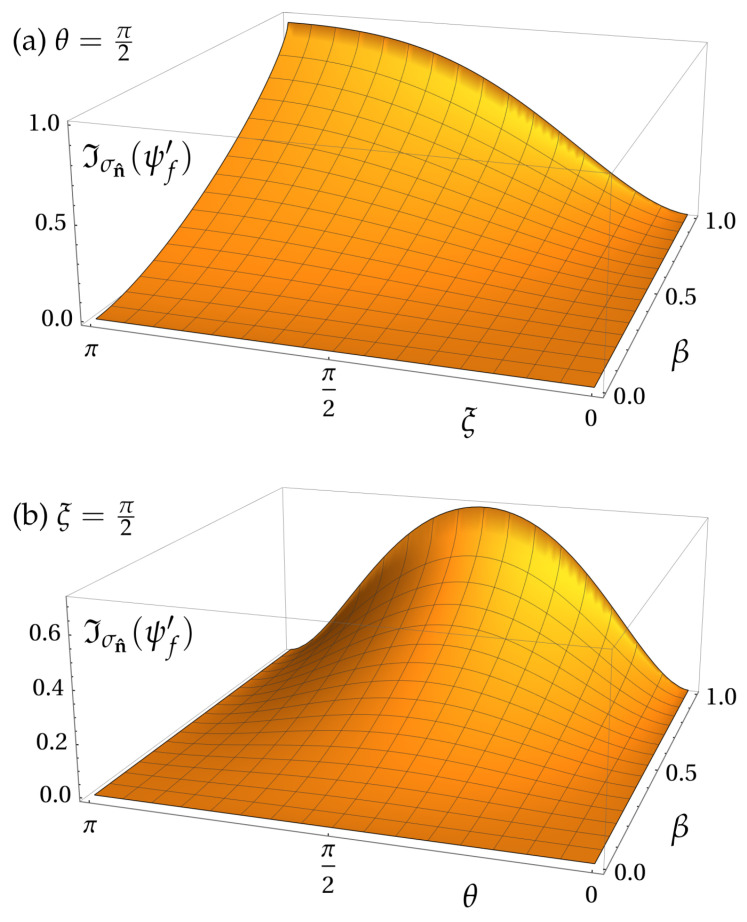
Irreality $I_{\sigma_{\hat{n}}}\left(\psi_{f}^{\prime }\right)$ of $\sigma_{\hat{n}}$ for the output state $\psi_{f}^{\prime }$ of the MZI, as seen by the boosted frame $S^{\prime }$. (**a**) The MZI phase is held constant, ξ=π/2, and $I_{\sigma_{\hat{n}}}$ is plotted as function of the boost velocity β and the spin polar angle θ. (**b**) The spin polar angle is held constant, θ=π/2, and $I_{\sigma_{\hat{n}}}\left(\rho^{\prime }\right)$ is plotted as function of the MZI phase ξ. Irreality is shown to be maximal when ξ=π, a value for which there is no entanglement. This is due to $\sigma_{\hat{n}}$-coherence being generated by the Wigner rotation.

Figure 3 shows the plot of this expression for the same regimes of parameters we used for entanglement in Figure 2. The dependence on θ shows the same behavior observed for that case: $\sigma_{z}$ eigenstates are unaffected by the Wigner rotation due to the specific geometric configuration considered here. The dependence on ξ is more interesting: while entanglement is maximized for ξ=π/2 and is zero for ξ=π, the irreality of $\sigma_{\hat{n}}$ is maximal for ξ=π, when only the vertical detector D_1_ is reached with probability 1. Since no correlations are present, all of the irreality, in this case, is due to $\sigma_{\hat{n}}$-coherence being generated by the rotation. In fact, for the relative entropy of coherence $C_{\sigma_{\hat{n}}}\left(\rho_{S}^{\prime }\right)=S\left(\Phi_{\hat{n}}\left(\rho_{S}^{\prime }\right)\right)-S\left(\rho_{S}^{\prime }\right)$, the term $S\left(\Phi_{\hat{n}}\left(\rho_{S}^{\prime }\right)\right)$ evaluates to the same expression (19), such that when there is no entanglement, that is, $E\left(\psi_{f}^{\prime }\right)=S\left(\rho_{S}^{\prime }\right)=0$, the spin irreality is fully attributed to coherence. This is further confirmed by computing $D_{\sigma_{\hat{n}}}\left(\psi_{f}^{\prime }\right)=I_{\sigma_{\hat{n}}}\left(\psi_{f}^{\prime }\right)-C_{\sigma_{\hat{n}}}\left(\rho_{S}^{\prime }\right)$, the one-way quantum discord associated with $\sigma_{\hat{n}}$, and verifying that it is exactly equal to entanglement [Equation (17)]. Thus, in this particular case of a pure state, the irreality of $\sigma_{\hat{n}}$ is the sum of entanglement and $\sigma_{\hat{n}}$-coherence, each of these portions being affected differently by the Wigner rotation. This result shows that quantum irreality may increase with a Lorentz boost even in scenarios where entanglement remains unaffected.

### 3.3. Boost Effects on Momentum Irreality

We may also assess the reality status of the momentum by assigning a Hilbert space to each of its components. Rewriting state (12) in a more complete notation, which makes explicit each component of the four-momentum, we have

(20) $$\begin{array}{c} |\psi_{f}\rangle =\left[\cos\left(\frac{\xi }{2}\right)|\frac{E_{p}}{c},p,0,0\rangle +\sin\left(\frac{\xi }{2}\right)|\frac{E_{p}}{c},0,p,0\rangle \right]|\Phi \rangle . \end{array}$$

We see that the energy and the *z* component are the same in both branches of the superposition, meaning they can be factorized and essentially ignored. The *x* and *y* components, on the other hand, are entangled with each other. Within the discretized framework we have been using, the only accessible states for the *x* and *y* components are ${|0\rangle }_{x(y)}$ and ${|p\rangle }_{x(y)}$, meaning that each component behaves as a qubit. The entanglement between these components reads $E\left(\psi_{f}\right)=h\left[\;\cos^{2}\left(\xi /2\right)\right]$, which is zero when ξ∈{0,π} and reaches the maximum value $E(\psi_{f})=1$ when ξ=π/2. Direct calculations show that the total irreality of each momentum component behaves in the same manner, that is, $I_{P_{x}}\left(\psi_{f}\right)=I_{P_{y}}\left(\psi_{f}\right)=h\left[\;\cos^{2}\left(\xi /2\right)\right]$. Hence, both components turn out to be elements of reality only when ξ∈{0,π}. As for the moving frame $S^{\prime }$, the transformed state (12) can also be written in terms of the momentum components with the aid of Equation (13):

$$\begin{array}{c} |\psi_{f}^{\prime }\rangle =\cos\left(\frac{\xi }{2}\right)|\pi_{H}^{0},\pi_{H}^{x},0,0\rangle |\Phi \rangle +\sin\left(\frac{\xi }{2}\right)|\pi_{V}^{0},\pi_{V}^{x},\pi_{V}^{y},0\rangle |\Phi_{R}\rangle . \end{array}$$

Here we notice something unexpected: while the *z* component remains unaffected by the boost, the zero-component (energy) is not the same for $\pi_{H}$ and $\pi_{V}$, showing that a Lorentz boost may also entangle a particle’s energy with its three-momentum, an effect which is rarely discussed in the literature of relativistic quantum information.

The irreality of $P_{x}$ and $P_{y}$ are both found to boost independent and equal to the same value as in the lab frame: $I_{P_{x}}\left(\psi_{f}^{\prime }\right)=I_{P_{y}}\left(\psi_{f}^{\prime }\right)=h\left[\;\cos^{2}\left(\xi /2\right)\right]$. Likewise, the entanglement between one of these momentum components and the rest of the degrees of freedom is also boosted independent and equal to $E\left(\psi_{f}^{\prime }\right)=S\left(\rho_{P_{x}}^{\prime }\right)=S\left(\rho_{P_{y}}^{\prime }\right)=h\left[\;\cos^{2}\left(\xi /2\right)\right]$. Energy—an element of reality in the lab frame—turns out to be an entangled degree of freedom violating reality in the moving frame, with an equal dependence of its irreality on ξ as the components $P_{x}$ and $P_{y}$. We notice that the irreality of all components of the four-momentum depends solely on the MZI phase ξ, that is, both reference frames agree that the momentum ontology is dictated solely by intrinsic characteristics of the interferometer. Now, there is, of course, no reason for one to believe that such absoluteness of the momentum irreality will manifest in more general contexts.

## 4. Discussion

In this work, we have taken the investigations of relativistic quantum information a step further by looking into how quantum irreality, as defined by Ref. [12], is affected by a Lorentz boost. In particular, we have verified that the spin irreality may increase with the boost velocity, even in situations where the entanglement is not affected by the transformation. This is because a Wigner rotation may affect spin coherence, even if there is no momentum superposition (and therefore no entanglement). Our approach also outlined a peculiar effect, often overlooked in the relativistic quantum information literature, namely, that the boost may also create entanglement between a particle’s energy and its other degrees of freedom. On the other hand, as far as momentum is concerned, we have shown, via an explicit case study, that irreality can be invariant under certain specific boosts.

All in all, our results show that quantum irrealism is not an absolute notion under Lorentz transformations. More specifically, this means that, as far as Bilobran and Angelo’s model of realism is concerned, observers in relative motion will generally disagree on their conclusions about the physical reality of the observables to which they have access. This viewpoint agrees with the findings of a recent study conducted within the context of Galilei transformations between quantum reference frames [59]. Nevertheless, it should be remarked that, under critical assessment, the relativity of physical reality cannot be decreed before the advance of the research toward the measurement of the relativistic particle’s spin. Most of the analyses realized so far admit that the messages derived from the formalism are correct, but it seems fair to say that we still have not reached a consensus about what the relativistic spin actually is [47,51,52,53]. This defines an interesting research program.

Besides contributing to deepening foundational perspectives in the interface between quantum mechanics and special relativity, we hope that our results will aid in the understanding of the relativity of quantum resources, which may become increasingly important the further we advance with quantum technologies.

## Data Availability

Not applicable.

## References

[1] Einstein A., Podolsky B., Rosen N. (1935). Can Quantum-Mechanical Description of Physical Reality Be Considered Complete?. Phys. Rev..

[2] Bohr N. (1935). Can Quantum-Mechanical Description of Physical Reality be Considered Complete?. Phys. Rev..

[3] Bohm D. (1952). A Suggested Interpretation of the Quantum Theory in Terms of “Hidden” Variables. I. Phys. Rev..

[4] Bohm D. (1952). A Suggested Interpretation of the Quantum Theory in Terms of “Hidden” Variables. II. Phys. Rev..

[5] Bell J.S. (1964). On the Einstein Podolsky Rosen paradox. Phys. Phys. Fiz..

[6] Hensen B., Bernien H., Dréau A.E., Reiserer A., Kalb N., Blok M.S., Ruitenberg J., Vermeulen R.F., Schouten R.N., Abellán C. (2015). Loophole-free Bell inequality violation using electron spins separated by 1.3 kilometres. Nature.

[7] Giustina M., Versteegh M.A.M., Wengerowsky S., Handsteiner J., Hochrainer A., Phelan K., Steinlechner F., Kofler J., Larsson J.A., Abellán C. (2015). Significant-Loophole-Free Test of Bell’s Theorem with Entangled Photons. Phys. Rev. Lett..

[8] Shalm L.K., Meyer-Scott E., Christensen B.G., Bierhorst P., Wayne M.A., Stevens M.J., Gerrits T., Glancy S., Hamel D.R., Allman M.S. (2015). Strong Loophole-Free Test of Local Realism. Phys. Rev. Lett..

[9] Hensen B., Kalb N., Blok M.S., Dréau A.E., Reiserer A., Vermeulen R.F., Schouten R.N., Markham M., Twitchen D.J., Goodenough K. (2016). Loophole-free Bell test using electron spins in diamond: Second experiment and additional analysis. Sci. Rep..

[10] Rauch D., Handsteiner J., Hochrainer A., Gallicchio J., Friedman A.S., Leung C., Liu B., Bulla L., Ecker S., Steinlechner F. (2018). Cosmic Bell Test Using Random Measurement Settings from High-Redshift Quasars. Phys. Rev. Lett..

[11] Li M.H., Wu C., Zhang Y., Liu W.Z., Bai B., Liu Y., Zhang W., Zhao Q., Li H., Wang Z. (2018). Test of Local Realism into the Past without Detection and Locality Loopholes. Phys. Rev. Lett..

[12] Bilobran A.L.O., Angelo R.M. (2015). A measure of physical reality. Europhys. Lett..

[13] Dieguez P.R., Guimarães J.R., Peterson J.P.S., Angelo R.M., Serra R.M. (2022). Experimental assessment of physical realism in a quantum-controlled device. Commun. Phys..

[14] Wigner E.P., Good I.J. (1961). Remarks on the Mind-Body Question. The Scientist Speculates.

[15] Frauchiger D., Renner R. (2018). Quantum theory cannot consistently describe the use of itself. Nat. Commun..

[16] Brukner C. (2018). A No-Go Theorem for Observer-Independent Facts. Entropy.

[17] Bong K.W., Utreras-Alarcón A., Ghafari F., Liang Y.C., Tischler N., Cavalcanti E.G., Pryde G.J., Wiseman H.M. (2020). A strong no-go theorem on the Wigner’s friend paradox. Nat. Phys..

[18] Horodecki R., Horodecki P., Horodecki M., Horodecki K. (2009). Quantum entanglement. Rev. Mod. Phys..

[19] Chitambar E., Gour G. (2019). Quantum resource theories. Rev. Mod. Phys..

[20] Gisin N., Ribordy G., Tittel W., Zbinden H. (2002). Quantum cryptography. Rev. Mod. Phys..

[21] Bennett C.H., Brassard G., Crépeau C., Jozsa R., Peres A., Wootters W.K. (1993). Teleporting an unknown quantum state via dual classical and Einstein–Podolsky-Rosen channels. Phys. Rev. Lett..

[22] Boschi D., Branca S., De Martini F., Hardy L., Popescu S. (1998). Experimental Realization of Teleporting an Unknown Pure Quantum State via Dual Classical and Einstein–Podolsky-Rosen Channels. Phys. Rev. Lett..

[23] Martín-Martínez E., Menicucci N.C. (2012). Cosmological quantum entanglement. Class. Quantum Grav..

[24] Marais A., Adams B., Ringsmuth A.K., Ferretti M., Gruber J.M., Hendrikx R., Schuld M., Smith S.L., Sinayskiy I., Krüger T.P.J. (2018). The future of quantum biology. J. R. Soc. Interface.

[25] Peres A., Terno D.R. (2004). Quantum information and relativity theory. Rev. Mod. Phys..

[26] Peres A., Scudo P.F., Terno D.R. (2002). Quantum Entropy and Special Relativity. Phys. Rev. Lett..

[27] Alsing P.M., Milburn G. (2002). Lorentz Invariance of Entanglement. Quantum Inf. Comput..

[28] Czachor M. (2005). Comment on “Quantum Entropy and Special Relativity”. Phys. Rev. Lett..

[29] Dunningham J., Palge V., Vedral V. (2009). Entanglement and nonlocality of a single relativistic particle. Phys. Rev. A.

[30] Saldanha P.L., Vedral V. (2012). Physical interpretation of the Wigner rotations and its implications for relativistic quantum information. New J. Phys..

[31] Saldanha P.L., Vedral V. (2013). Wigner rotations and an apparent paradox in relativistic quantum information. Phys. Rev. A.

[32] Taillebois E.R.F., Avelar A.T. (2013). Spin-reduced density matrices for relativistic particles. Phys. Rev. A.

[33] Zambianco M.H., Landulfo A.G.S., Matsas G.E.A. (2019). Observer dependence of entanglement in nonrelativistic quantum mechanics. Phys. Rev. A.

[34] Bittencourt V.A.S.V., Blasone M. (2020). Single particle entanglement of a massive relativistic particle: Dirac bispinors and spin 1/2 states. J. Phys. Conf. Ser..

[35] Bernardini A.E., Bittencourt V.A.S.V., Blasone M. (2020). Lorentz invariant quantum concurrence for *SU*(2) ⊗ *SU*(2) spin–parity states. Eur. Phys. J. Plus.

[36] Gingrich R.M., Adami C. (2002). Quantum Entanglement of Moving Bodies. Phys. Rev. Lett..

[37] Jordan T.F., Shaji A., Sudarshan E.C.G. (2007). Lorentz transformations that entangle spins and entangle momenta. Phys. Rev. A.

[38] Chakrabarti A. (2009). Entangled states, Lorentz transformations and spin precession in magnetic fields. J. Phys. A Math. Theor..

[39] Friis N., Bertlmann R.A., Huber M., Hiesmayr B.C. (2010). Relativistic entanglement of two massive particles. Phys. Rev. A.

[40] Choi T., Hur J., Kim J. (2011). Relativistic effects on the spin entanglement of two massive Dirac particles. Phys. Rev. A.

[41] Palge V., Dunningham J. (2015). Behavior of Werner states under relativistic boosts. Ann. Phys..

[42] Palge V., Dunningham J., Groote S., Liivat H. (2018). Relativistic entanglement of two particles driven by continuous product momenta. Phys. Rev. A.

[43] Bittencourt V.A.S.V., Bernardini A.E., Blasone M. (2018). Global Dirac bispinor entanglement under Lorentz boosts. Phys. Rev. A.

[44] Fan J., Li X. (2018). Relativistic effect of entanglement in fermion-fermion scattering. Phys. Rev. D.

[45] Petreca A.T., Cardoso G., Devecchi F.P., Angelo R.M. (2022). Genuine multipartite entanglement and quantum coherence in an electron-positron system: Relativistic covariance. Phys. Rev. A.

[46] Czachor M. (1997). Einstein–Podolsky-Rosen-Bohm experiment with relativistic massive particles. Phys. Rev. A.

[47] Lee D., Chang-Young E. (2004). Quantum entanglement under Lorentz boost. New J. Phys..

[48] Caban P., Rembieliński J. (2005). Lorentz-covariant reduced spin density matrix and Einstein–Podolsky-Rosen–Bohm correlations. Phys. Rev. A.

[49] Kim W.T., Son E.J. (2005). Lorentz-invariant Bell’s inequality. Phys. Rev. A.

[50] Streiter L.F., Giacomini F., Brukner C. (2021). Relativistic Bell Test within Quantum Reference Frames. Phys. Rev. Lett..

[51] Bauke H., Ahrens S., Keitel C.H., Grobe R. (2014). Relativistic spin operators in various electromagnetic environments. Phys. Rev. A.

[52] Bauke H., Ahrens S., Keitel C.H., Grobe R. (2014). What is the relativistic spin operator?. New J. Phys..

[53] Giacomini F., Castro-Ruiz E., Brukner C. (2019). Relativistic Quantum Reference Frames: The Operational Meaning of Spin. Phys. Rev. Lett..

[54] Gonera C., Kosiński P., Maślanka P. (2004). Special relativity and reduced spin density matrices. Phys. Rev. A.

[55] Thomas L.H. (1926). The Motion of the Spinning Electron. Nature.

[56] Wigner E. (1939). On Unitary Representations of the Inhomogeneous Lorentz Group. Ann. Math..

[57] O’Donnell K., Visser M. (2011). Elementary analysis of the special relativistic combination of velocities, Wigner rotation and Thomas precession. Eur. J. Phys..

[58] Giacomini F., Castro-Ruiz E., Brukner C. (2019). Quantum mechanics and the covariance of physical laws in quantum reference frames. Nat. Commun..

[59] Savi M.F., Angelo R.M. (2021). Quantum resource covariance. Phys. Rev. A.

[60] Gomes V.S., Angelo R.M. (2018). Nonanomalous measure of realism-based nonlocality. Phys. Rev. A.

[61] Fucci D.M., Angelo R.M. (2019). Tripartite realism-based quantum nonlocality. Phys. Rev. A.

[62] Orthey A.C., Angelo R.M. (2019). Nonlocality, quantum correlations, and violations of classical realism in the dynamics of two noninteracting quantum walkers. Phys. Rev. A.

[63] Dieguez P.R., Angelo R.M. (2018). Information-reality complementarity: The role of measurements and quantum reference frames. Phys. Rev. A.

[64] Engelbert N.G., Angelo R.M. (2020). Hardy’s paradox as a demonstration of quantum irrealism. Found. Phys..

[65] Freire I.S., Angelo R.M. (2019). Quantifying continuous-variable realism. Phys. Rev. A.

[66] Orthey A.C., Angelo R.M. (2022). Quantum realism: Axiomatization and quantification. Phys. Rev. A.

[67] Costa A.C.S., Angelo R.M. (2020). Information-based approach towards a unified resource theory. Quantum Inf. Process..

[68] Baumgratz T., Cramer M., Plenio M.B. (2014). Quantifying Coherence. Phys. Rev. Lett..

[69] Weinberg S. (1995). The Quantum Theory of Fields.

[70] Pereira S.T., Angelo R.M. (2015). Galilei covariance and Einstein’s equivalence principle in quantum reference frames. Phys. Rev. A.

